# Role of *AT1G72910*, *AT1G72940*, and *ADR1-LIKE* *2* in Plant Immunity under Nonsense-Mediated mRNA Decay-Compromised Conditions at Low Temperatures

**DOI:** 10.3390/ijms21217986

**Published:** 2020-10-27

**Authors:** Zeeshan Nasim, Muhammad Fahim, Katarzyna Gawarecka, Hendry Susila, Suhyun Jin, Geummin Youn, Ji Hoon Ahn

**Affiliations:** 1 Department of Life Sciences, Korea University, Seoul 02841, Korea; znasim09@korea.ac.kr (Z.N.); katgaw@korea.ac.kr (K.G.); susila_hendry@korea.ac.kr (H.S.); letgo1003@korea.ac.kr (S.J.); youngm32@korea.ac.kr (G.Y.); 2 Centre for Omic Sciences, Islamia College University, Peshawar 25120, Pakistan; fahim@icp.edu.pk

**Keywords:** NMD, Arabidopsis, immunity, NLRs, salicylic acid, temperature

## Abstract

Nonsense-mediated mRNA decay (NMD) removes aberrant transcripts to avoid the accumulation of truncated proteins. NMD regulates nucleotide-binding, leucine-rich repeat (NLR) genes to prevent autoimmunity; however, the function of a large number of NLRs still remains poorly understood. Here, we show that three NLR genes (*AT1G72910*, *AT1G72940*, and *ADR1-LIKE 2*) are important for NMD-mediated regulation of defense signaling at lower temperatures. At 16 °C, the NMD-compromised *up-frameshift protein1* (*upf1*) *upf3* mutants showed growth arrest that can be rescued by the artificial miRNA-mediated knockdown of the three NLR genes. mRNA levels of these NLRs are induced by *Pseudomonas syringae* inoculation and exogenous SA treatment. Mutations in *AT1G72910*, *AT1G72940*, and *ADR1-LIKE 2* genes resulted in increased susceptibility to *Pseudomonas syringae*, whereas their overexpression resulted in severely stunted growth, which was dependent on basal disease resistance genes. The NMD-deficient *upf1 upf3* mutants accumulated higher levels of NMD signature-containing transcripts from these NLR genes at 16 °C. Furthermore, mRNA degradation kinetics showed that these NMD signature-containing transcripts were more stable in *upf1 upf3* mutants. Based on these findings, we propose that *AT1G72910*, *AT1G72940*, and *ADR1-LIKE 2* are directly regulated by NMD in a temperature-dependent manner and play an important role in modulating plant immunity at lower temperatures.

## 1. Introduction

Plants integrate external cues, including biotic/abiotic stresses, light, and temperature during growth and development. Among these environmental cues, temperature affects plant growth, development, and immunity [1]. Temperature and plant growth interact in an antagonistic manner: at higher ambient temperatures, growth is favored while immunity is generally compromised [2]. For example, Arabidopsis plants grown at 30 °C are less able to respond to (and restrict the growth of) bacterial pathogens compared to plants grown at 23 °C [2]. The negative association between plant immunity and ambient temperature may involve the transcriptional regulation of genes involved in plant growth [3], but the role of post-transcriptional regulation remains to be explored.

In one type of post-transcriptional regulation, eukaryotes use nonsense-mediated mRNA decay (NMD) to eliminate aberrant mRNAs from cells, thus preventing the accumulation of truncated proteins. Key features of NMD targets include premature termination codons (PTCs) generated by mutations, transcriptional errors, or alternative splicing (AS) events [4]. The core NMD machinery includes the conserved proteins UP-FRAMESHIFT PROTEIN1 (UPF1), UPF2, and UPF3, which recognize targets of NMD [5]. Genome-wide transcriptome analyses showed that transcripts containing upstream open reading frames (uORFs), long 3′ untranslated regions (UTRs), and introns in 3′ UTRs are targeted by NMD and that ~21% of endogenous transcripts contain such signatures [6,7]. Consistent with this global regulation, impairment of NMD causes various developmental defects, including stunted growth, abnormal leaf morphology, altered flowering time, and enhanced resistance to bacterial infection [8,9].

The connection between plant immunity and ambient temperature also likely involves plant hormones, which play key roles in numerous growth/developmental processes and mediate defense responses against diverse biotic and abiotic stresses [10]. The plant hormones salicylic acid (SA), jasmonic acid (JA), and ethylene (ET) play critical roles in plant defense against biotic stress. The levels of these hormones start to increase upon pathogen infection [10]. SA mediates defense responses against biotrophic pathogens, whereas the JA and ET pathways primarily function in plant responses to necrotrophic pathogens [11]. In Arabidopsis, two distinct pathways are involved in SA biosynthesis, i.e., the ICS pathway (involving ISOCHORISMATE SYNTHASE1 [ICS1]) and the PAL pathway (involving PHENYLALANINE AMMONIA LYASE [PAL]), both of which use the same precursor, chorismite. The ICS pathway predominantly contributes to overall SA biosynthesis, as the *ICS1*-defective *sid2* mutant synthesizes only ~10% of wild-type levels of total SA [12]. However, inhibiting the PAL pathway also significantly decreases SA levels [13]. SA triggers the monomerization of NONEXPRESSOR OF PR GENES1 (NPR1), leading to its phosphorylation, which is critical for its nuclear localization, and inducing the expression of defense genes that act downstream of SA [14,15].

The plant immune system has successive layers of defense to restrict pathogen infection. One layer uses plasma membrane-localized receptors that detect conserved pathogen-associated molecular patterns (PAMPs). PAMP recognition induces transcriptional reprogramming, referred to as PAMP-triggered immunity (PTI) [16]. However, virulent pathogens interfere with host defense programs by secreting various effectors. ENHANCED DISEASE SUSCEPTIBILITY1 (EDS1) functions with the related protein PHYTOALEXIN DEFICIENT4 (PAD4) in regulating the basal defense layer and thus restricts the spread of pathogens, in part via the SA defense signaling pathway [17]. Basal resistance is further reinforced by intracellular nucleotide-binding, leucine-rich repeat (NLR) receptors, which detect specific pathogen effector molecules to confer effector-triggered immunity (ETI) [18]. ETI employs components of the basal resistance machinery to enhance plant defense, often resulting in programmed cell death (PCD), which is also referred to as the hypersensitive response (HR) [19].

Plant NLRs play an important role in pathogen detection. In the absence of the effector, NLRs are thought to exist in equilibrium between the ON and OFF states. When the effector is present, it binds to and stabilizes NLRs in the ON state, thus shifting the equilibrium toward an active form to trigger defense responses [20]. NLR gene activity is also regulated at the transcriptional and post-transcriptional levels [21], and NLR misexpression triggers autoimmunity characterized by severe stunting and fitness costs [22]. In addition, AS-coupled NMD constitutes another layer of NLR regulation. AS can produce NLR transcripts containing NMD signatures that are cleared out by NMD, which is consistent with the finding that a number of NLR genes act as NMD substrates [23,24].

Although NMD is a common feature of the global surveillance system in plants and is known to modulate plant immunity via post-transcriptional and post-translational regulation of NLR genes [23,24], a large number of NLR genes are functionally uncharacterized. Here, we report that a subgroup of NLR genes (*AT1G72910*, *AT1G72940*, and *ADR1-LIKE 2*) are directly regulated by NMD in a temperature-dependent manner and are important for the NMD-mediated regulation of defense signaling at lower temperatures.

## 2. Results

### 2.1. The NMD-Deficient upf1 upf3 Mutants Exhibit Autoimmunity at 16 °C

**Figure 1:**
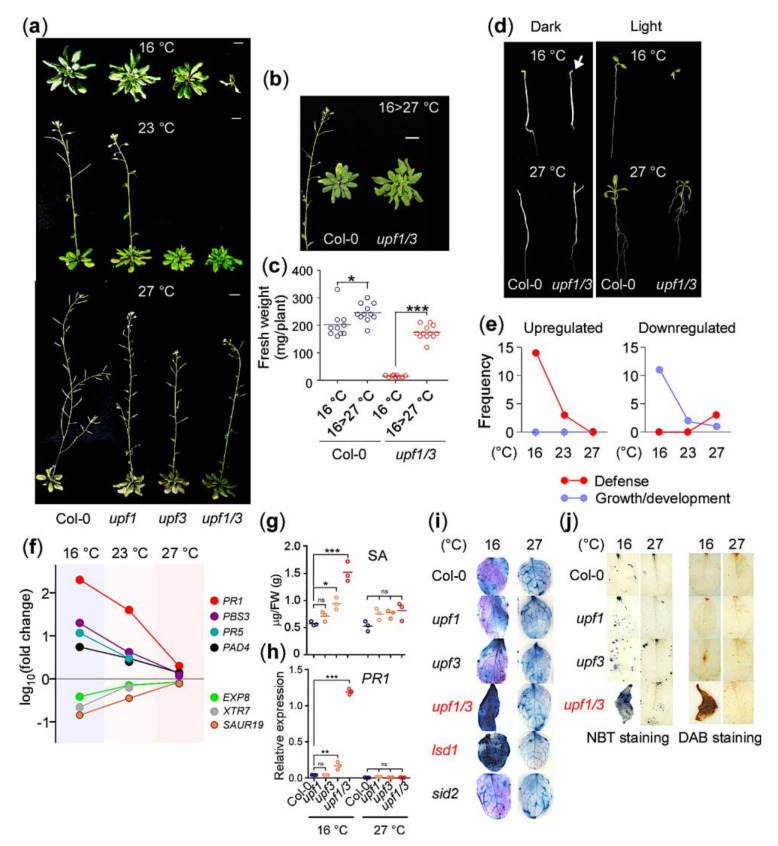
NMD (nonsense-mediated mRNA decay)-deficient *upf1/3* mutants show growth arrest at 16 °C (**a**) Phenotypes of *upf* mutants grown at 16 °C, 23 °C, and 27 °C in soil (scale bar = 1 cm). (**b**,**c**) Growth phenotypes (**b**) and fresh weight (**c**) of *upf1/3* mutants shifted from 16 °C to 27 °C. The photographs were taken 2 weeks after the temperature shift. (**d**) Phenotypes of *upf1/3* mutants grown with or without light (long days, 16:8 h light:dark) at 16 °C and 27 °C. (**e**) Gradual increase in the number of defense-related GO terms among upregulated genes and growth/development-related GO terms among downregulated genes in *upf1/3* mutants at lower temperatures. (**f**) Expression levels of marker genes of defense and growth/development in *upf1/3* mutants at 16 °C, 23 °C, and 27 °C. The expression levels are represented as log fold-change values from RNA-seq data. (**g**) Salicylic acid (SA) levels in *upf1/3* mutants at 16 °C and 27 °C compared with wild type (Col-0) plants. (**h**) Transcript levels of *PR1* in *upf* mutants at 16 °C and 27 °C. (**i**) Trypan blue staining of the leaves of *upf* mutants grown at 16 °C and 27 °C. Note that only leaves of *upf1/3* and *lsd1-2* mutants showed dark staining. SA-hyperaccumulating *lsd1* mutants and SA biosynthesis-defective *sid2* mutants were used as controls. (**j**) NBT (nitro blue tetrazolium) staining and DAB (3,3′-diaminobenzidine) staining assays using *upf1/3* mutants at 16 °C and 27 °C. *: *p* < 0.05; **: *p* < 0.01; ***: *p* ≤ 0.001; ns: not significant cycle.

To investigate the role of NMD across a range of ambient temperatures, we analyzed the phenotypes of *upf* mutants grown at 16 °C, 23 °C, and 27 °C. At 16 °C, *upf1-5 upf3-1* double mutants (hereafter *upf1/3*) showed a severe growth arrest phenotype after generating two cotyledons and a few leaf-like structures, although the *upf1-5* (hereafter *upf1*) and *upf3-1* (hereafter *upf3*) single mutants successfully completed their life cycle (Figure 1a). In contrast, the *upf1/3* double mutants showed only mild growth defects at 23 °C and 27 °C. The *upf1* and *upf3* single mutants grown at any of the tested temperatures did not show such severe growth arrest [9,25,26]. We also found that the arrested growth of *upf1/3* mutants could be rescued by shifting the temperature from 16 °C to 27 °C. Two weeks after transfer to 27 °C, the *upf1/3* mutants resumed growth and completed their life cycles (Figure 1b) and the fresh weight of *upf1/3* mutants shifted to 27 °C and was restored to the levels of wild-type plants (Figure 1c), which is consistent with previous reports that the growth defects in NMD-deficient mutants can be rescued by high temperature [9,23]. As light is an important environmental factor that affects plant growth and development, we also examined whether light affected the phenotype of *upf1/3* mutants. Notably, dark-grown *upf1/3* mutants did not show the growth arrest phenotype at 16 °C (arrow in Figure 1d), and neither did wild-type plants (Figure 1d).

To explore the mechanism underlying growth arrest of the *upf* mutants at 16 °C, we performed RNA-seq analyses using the *upf* mutants grown at 16 °C and 27 °C and compared the resulting data with our published transcriptome data of *upf* mutants at 23 °C (GSE87851) [26]. A large number of genes were differentially expressed in upf mutants at all tested temperatures (Supplementary Figure S1a–c), which is consistent with the published transcriptome data of NMD-deficient mutants [24,27,28]. Functional classification of the differentially expressed genes (DEGs) in *upf1/3* mutants revealed that GO terms related to defense, such as immune response, were significantly enriched in upregulated genes at 16 °C (Supplementary Figure S1d) [27]. In contrast, GO terms related to growth/development (such as epidermis development) were significantly enriched among downregulated genes in *upf1/3* mutants at 16 °C (Supplementary Figure S1e). We listed the top 20 highly enriched GO terms in *upf1/3* mutants at each temperature (Supplementary Figure S1d,e) and classified them into three categories: defense, growth/development, and others. For upregulated genes in the *upf1/3* mutants, 14, 3, and 0 terms related to defense were identified at 16 °C, 23 °C, and 27 °C, respectively, whereas no GO terms related to growth/development were identified at any temperature (Figure 1e). For downregulated genes in *upf1/3* mutants, 11, 2, and 1 terms related to growth/development were identified at 16 °C, 23 °C, and 27 °C, respectively.

We then measured the mRNA levels of defense and growth/development marker genes in *upf1/3* mutants. At 16 °C, we detected a dramatic increase in transcript levels of defense marker genes (*PATHOGENESIS-RELATED1* [*PR1*], *PR5*, *AVRPPHB SUSCEPTIBLE3* [*PBS3*], and *PHYTOALEXIN DEFICIENT4* [*PAD4*]) and a gradual decrease in transcript levels of growth/development marker genes (*EXPANSIN A8* [*EXP8*], *XYLOGLUCAN ENDOTRANSGLYCOSYLASE7* [*XTR7*], and *SMALL AUXIN UP RNA19* [*SAUR19*]) (Figure 1f). These antagonistic expression patterns of genes regulating defense responses and growth/development suggested that a substantial proportion of resources are allocated to defense at the expense of growth/development if NMD is impaired at lower temperatures. These results highlight the importance of functional NMD in regulating the balance between immunity and growth/development in a temperature-dependent manner.

NMD-deficient mutants accumulate higher levels of SA [8,9]. Quantification of SA in upf mutants confirmed significant SA accumulation at 16 °C only (Figure 1g). Moreover, the mRNA levels of *PR1* were dramatically induced in *upf1/3* mutants at 16 °C only (Figure 1h). Trypan blue staining resulted in strong dark-blue staining of *upf1/3* mutant leaves at 16 °C, suggesting that almost all leaf cells were dead (Figure 1i). A similar staining pattern was observed in *lsd1*-2 mutants at 16 °C, but *sid2-1* mutants did not show strong staining at any temperature. These results suggested that strong hypersensitive response (HR) was elicited in *upf1/3* mutants at 16 °C, eventually leading to death. We also performed nitro blue tetrazolium (NBT) staining and 3,3′-diaminobenzidine (DAB) staining assays to detect superoxide and H_2_O_2_, respectively, to investigate whether the interplay between SA and reactive oxygen species (ROS) occurs in *upf1/3* mutants [29,30]. We found strongly stained leaves in *upf1/3* mutants at 16 °C, indicating that excess superoxide and H_2_O_2_ are produced in *upf1/3* mutants at 16 °C (Figure 1j). The elevated levels of SA in the *upf* mutants are due to the upregulation of *ICS1*, *PAL1, 2* and *3*, the SA biosynthesis genes (Supplementary Figure S2a). Treating the *upf1/3* plants with 1-aminobenzotriazole (ABT), which inhibits the PAL pathway of SA biosynthesis, or introducing mutation in SA biosynthetic enzyme *ICS1*/*SID2*, rescued the HR of *upf1/3* mutants grown at 16 °C (Supplementary Figure S2b,c). Moreover, blocking NLR signaling by introducing mutation in *EDS1*, which encodes a basal/TLN-NLR immunity regulator, also rescued the severely stunted phenotype and *PR1* expression (Supplementary Figure S2d,e) [23]. Taken together, these results suggest that NMD deficiency results in elicitation of a strong temperature-dependent autoimmune response that causes imbalance between growth/development and defense [23,24].

### 2.2. Identification of AT1G72910, AT1G72940, and ADR1-LIKE 2 as NLR Genes Regulated by NMD at 16 °C

**Figure 2:**
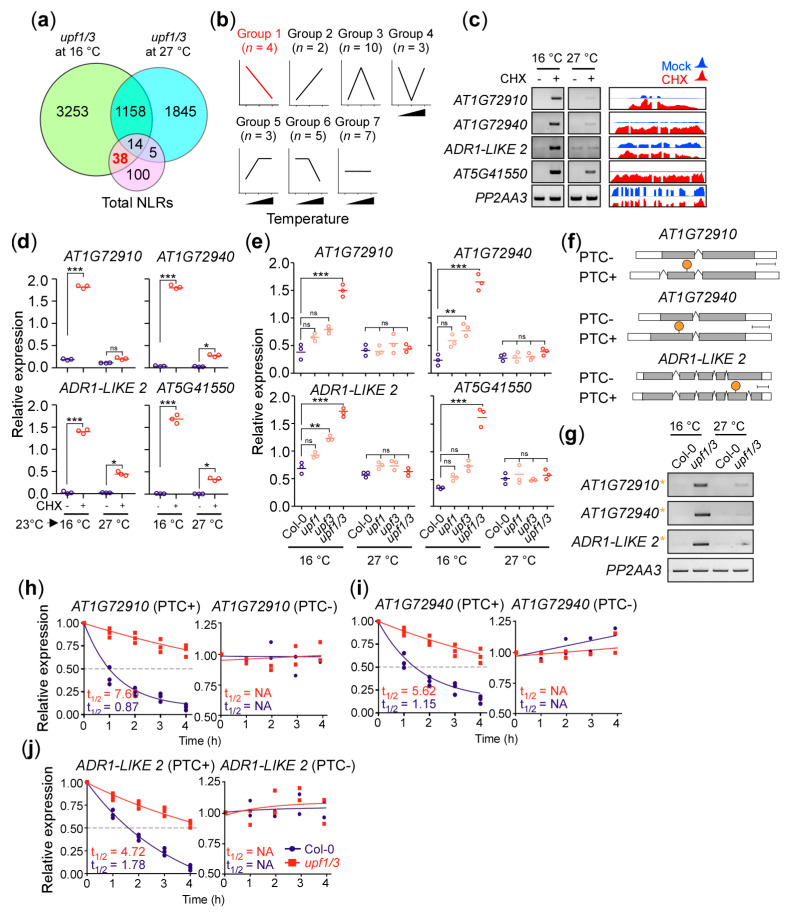
Identification of *AT1G72910*, *AT1G72940*, and *ADR1-LIKE 2* as NLR (nucleotide-binding, leucine-rich repeat) genes regulated by NMD in a temperature-dependent manner. (**a**) Venn diagram highlighting the 38 NLR genes that are upregulated in *upf1/3* mutants compared with wild type (Col-0) at 16 °C. (**b**) Classification of the 38 NLR genes into seven groups based on their expression patterns in CHX-treated wild-type plants at 16 °C, 23 °C, and 27 °C. *n* = the number of NLR genes in each group. (**c**) Expression levels of group 1 NLR genes determined by RT-PCR. Read coverage graphs from the public transcriptome data (GSE41432) upon CHX treatment are shown on the right. (**d**) qPCR confirmation of the group 1 NLR genes in CHX-treated wild-type plants. Seedlings were grown at 23 °C and transferred to each temperature for CHX treatment. (**e**) Expression levels of group 1 NLR genes in the *upf* mutants at 16 °C and 27 °C. (**f**) Map of transcripts with a PTC (PTC+) and without a PTC (PTC−) identified from the *AT1G72910*, *AT1G72940*, and *ADR1-LIKE 2* loci. A PTC is indicated by an orange circle. (**g**) Confirmation of the presence of PTC+ transcripts (orange asterisk) from *AT1G72910*, *AT1G72940*, and *ADR1-LIKE 2* in *upf1/3* mutants at 16 °C and 27 °C via RT-PCR. *PP2AA3* was used as an internal control. (**h**) mRNA degradation kinetics of the PTC+/− transcripts of *AT1G72910*, (**i**) *AT1G72940*, and (**j**) *ADR1-LIKE 2* in *upf1/3* mutants. The half-lives of PTC+ transcripts shown for Col-0 and *upf1/3* mutants. One-way ANOVA with post-hoc Tukey test was used for the statistical comparison of all genotypes. *: *p* < 0.05; **: *p* < 0.01, ***: *p* ≤ 0.001; ns: not significant. NA: not available.

The facts that (1) SA signaling is downstream of NLRs [31,32], (2) repression of basal/TLN-NLR immunity resulted in phenotypic rescue of *upf1/3* mutants (Supplementary Figure S2d) [9], and (3) NLRs are regulated by NMD [23], prompted us to check if we can identify some NLR genes that show upregulation in *upf1/3* mutants at 16 °C. Analysis of *upf1/3* transcriptome data revealed 38 NLR genes that are upregulated in *upf1/3* mutants specifically at 16 °C (Figure 2a, Supplementary Table S1). We then analyzed the expression patterns of the 38 NLR genes via conventional RT-PCR at 16 °C, 23 °C, and 27 °C to further narrow down the list of candidate genes. For these experiments, we first confirmed CHX-induced NMD inhibition by measuring the levels of larger splice variants of *AT2G42500*, a known NMD target [6] (Supplementary Figure S3a). The NLR genes were then classified into seven groups based on their changes in expression levels in CHX-treated seedlings (Figure 2b). For instance, group 1 NLR genes include *AT1G72910*, *AT1G72940*, *AT5G41550*, and *ADR1-LIKE 2*, whose expression levels increased at 16 °C after CHX treatment (Figure 2c). Their read coverage in CHX-treated wild-type plants (GSE41432) is shown in Figure 2c and RT-PCR results and read coverage graphs of genes in the remaining groups are shown in Supplementary Figure S3b. We further tested the temperature-dependent accumulation of group 1 NLR transcripts in CHX-treated plants via qPCR analyses (Figure 2d). Consistent with the observation, expression levels of the group 1 NLR genes were altered in *upf1/3* mutants at 16 °C (Figure 2e), suggesting them to be the potential targets of NMD.

To test whether these group 1 NLR genes are direct targets of NMD, we searched our RNA-seq data for any of their transcripts that contained potential NMD signatures. We found out-of-frame transcripts containing a PTC from three of these NLR genes (Figure 2f); however, we could not detect any NMD signature-containing transcript of *AT5G41550*. To further confirm, we performed conventional RT-PCR and found that their PTC+ transcripts were predominantly expressed in *upf1/3* mutants (Figure 2g), suggesting that these transcripts are indeed the targets of NMD.

To test whether *AT1G72910*, *AT1G72940*, and *ADR1-LIKE 2* are direct targets of NMD, we carried out mRNA degradation kinetics analyses to test the stability of their transcripts after treatment with the transcriptional inhibitor cordycepin [33]. We observed increased half-lives for PTC+ transcripts from *AT1G72910*, *AT1G72940*, and *ADR1-LIKE 2* in *upf1/3* mutants (Figure 2h–j). For instance, PTC+ transcripts of *AT1G72910* had a half-life of ~7.5 h in *upf1/3* mutants, compared to ~0.8 h in wild-type plants. These observations suggested that NMD fine-tunes the transcript levels of *AT1G72910*, *AT1G72940*, and *ADR1-LIKE 2* in a temperature-dependent manner to prevent the elicitation of immunity in the absence of invading pathogens.

### 2.3. AT1G72910, AT1G72940, and ADR1-LIKE 2 Are Required for Pathogen Response at Lower Temperatures

**Figure 3:**
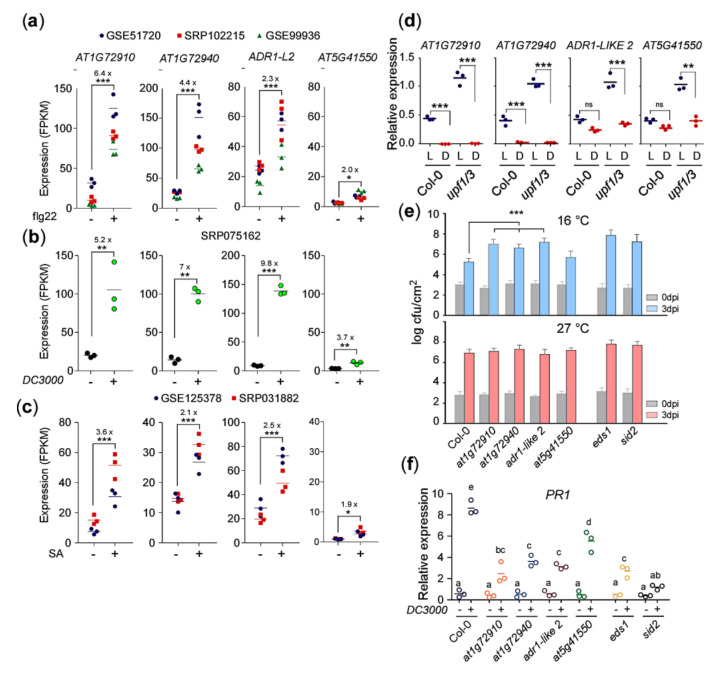
*AT1G72910*, *AT1G72940*, and *ADR1-LIKE 2* are important for plant immunity. (**a**) Expression of group 1 NLR genes in WT plants in response to flg22 treatment, (**b**) bacterial inoculation, and (**c**) exogenous SA treatment. (**d**) Decrease in *AT1G72910*, *AT1G72940*, and *ADR1-LIKE 2* transcript levels in *upf1/3* mutants by darkness at 16 °C. L: light, D: dark. Increased susceptibility to bacterial pathogen *Pst* DC3000 (**e**) and decreased *PR1* mRNA levels (**f**) seen in *at1g72910, at1g72940*, and *adr1-like 2* mutants at 16 °C. *eds1* and *sid2* mutants were used as positive controls. dpi: days post-inoculation. Letters indicate significant difference from one-way ANOVA followed by Tukey’s range tests (*p* < 0.05) *: *p* < 0.05; **: *p* < 0.01, ***: *p* ≤ 0.001; ns: not significant.

Treatment of flg22, a synthetic 22-amino-acid domain in the N terminus of the bacterial flagellin, induces the expression of a number of NLRs, resulting in an antibacterial response [34]. To check whether the expression of the group 1 NLRs are also induced by flg22 treatment, we analyzed three public RNA-seq datasets of flg22-treated WT plants (GSE51720, SRP102215, GSE99936; Supplementary Table S2). The transcriptome analysis showed strong induction (*p*-value < 0.001) of *AT1G72910*, *AT1G72940*, and *ADR1-LIKE 2* genes in response to flg22 treatment (Figure 3a), whereas *AT5G41550* showed only a moderate induction (Figure 3a). A similar expression pattern was observed in the plants challenged with *Pseudomonas syringae* (SRP075162, Supplementary Table S2). *Pseudomonas syringae* infection induced the expression of the *AT1G72910*, *AT1G72940*, *ADR1-LIKE 2*, and *AT5G41550* genes by 5.2-, 7.0-, 9.8- and 3.7-fold, respectively (Figure 3b). The pathogen infection results in the accumulation of SA [35], which is known to induce the expression of NLR genes in a positive feedback loop [36,37,38]. Therefore, to check the effect of exogenous SA on the expression of these group 1 NLR genes, we analyzed two public transcriptome datasets of SA-treated WT plants (GSE125378, SRP031882, Supplementary Table S2). Consistent with the flg22-treated and bacteria-challenged WT plants, the exogenous SA treatment also induced the expression of group 1 NLR genes (Figure 3c). These results suggest that the expression of these group 1 NLRs, especially *AT1G72910*, *AT1G72940*, and *ADR1-LIKE 2*, are strongly induced upon pathogen infection and that they might be important for the response to pathogens.

Light is a key factor in regulating growth and development of the plant and its response to a number of environmental stimuli, including defense [39]. It is essential for SA accumulation and response to bacterial pathogens [40]. Furthermore, we observed that darkness rescued the growth arrest phenotype of *upf1/3* mutants at 16 °C (Figure 1d), which raised the possibility that the expression levels of group 1 NLR genes were affected by darkness. Indeed, qPCR analyses revealed that *AT1G72910* expression was downregulated dramatically (~73-fold) in *upf1/3* mutants in the dark (Figure 3d). In addition, the expression levels of *AT1G72940*, *ADR1-LIKE 2*, and *AT5G41550* genes were also reduced by ~36-, ~3-, and ~2.5-fold respectively, in the dark. These results suggested that light is required for expression of *AT1G72910*, *AT1G72940*, *ADR1-LIKE 2*, and *AT5G41550* to ensure the timely elicitation of plant defense responses.

To validate whether the group 1 NLR genes play a role in defense responses, we isolated T-DNA mutants of these NLR genes and challenged them with *Pst DC3000* at 16 °C and 27 °C, as induction of these NLR genes under NMD-impaired conditions might indicate their involvement in spontaneous activation of defense responses. We found that the mutant lines for *AT1G72910* (SALK_064034), *AT1G72940* (SALK_109730), and *ADR1-LIKE 2* (SALK_056402) were significantly more susceptible to *Pst DC3000* inoculation at 16 °C, compared to Col-0 plants (Figure 3e). Their susceptibility was similar to that seen in *eds1* and *sid2* mutants, which were used as positive controls. In contrast, a T-DNA mutant for *AT5G41550* (SALK_040476) showed similar resistance to Col-0 plants at 3 dpi (Figure 3e). At 27 °C, all genotypes showed similar susceptibility to the bacterial pathogen. Consistent with the increased susceptibility seen in *at1g72910*, *at1g72940*, and *adr1-like 2* mutants, the induction of *PR1* expression was reduced in these three NLR mutants at 16 °C (Figure 3f). Overall, these results suggested that *AT1G72910*, *AT1G72940*, and *ADR1-LIKE 2* are important for the induction of defense in response to bacterial pathogens in *upf1/3* mutants at 16 °C. As *at5g41550* mutants showed comparable bacterial growth with wild-type plants at 16 °C (Figure 3e) and a weak decrease in *PR1* mRNA levels (Figure 3f), we excluded *AT5G41550* from further analyses.

### 2.4. Knockdown of AT1G72910, AT1G72940, and ADR1-LIKE 2 Can Rescue the Growth Arrest Phenotype of upf1/3 Mutants

**Figure 4:**
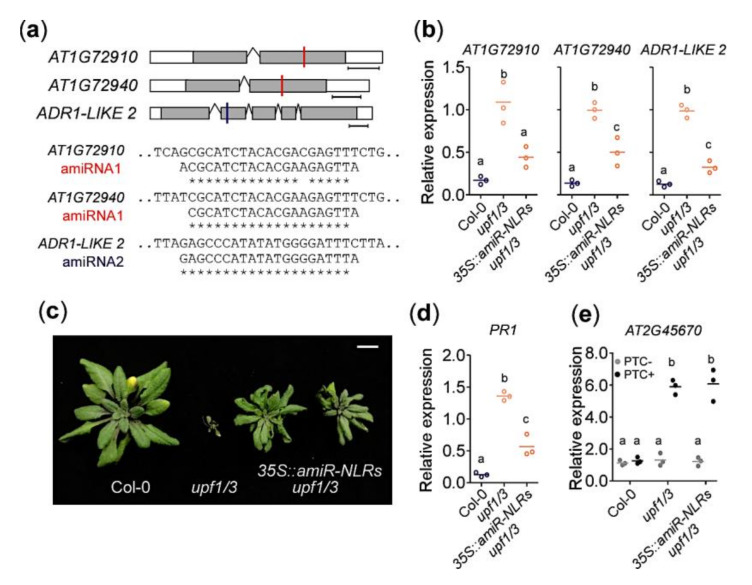
*AT1G72910*, *AT1G72940*, and *ADR1-LIKE 2* are NLR genes responsible for the severe growth defects of *upf1/3* mutants at 16 °C. (**a**) Sequences of amiRNAs. amiRNA1 is designed to target both *AT1G72910* and *AT1G72940*, whereas amiRNA2 is designed to target *ADR1-LIKE 2*. Target sites of amiRNA1 and amiRNA2 are indicated by red and blue lines, respectively. Note that reverse complementary sequences of amiRNA are shown. (**b**) Decrease in expression levels of *AT1G72910*, *AT1G72940*, and *ADR1-LIKE 2* by amiRNAs in *35S::amiR-NLRs upf1/3* plants. (**c**) Escape from growth arrest seen in *35S::amiR-NLRs upf1/3* plants at 16 °C. (**d**) Decrease in *PR1* expression levels in *35S::amiR-NLRs upf1/3* plants. (**e**) Increased levels of PTC+ transcripts of *AT2G45670* in *35S::amiR-NLRs upf1/3* plants. Letters indicate significant difference from one-way ANOVA followed by Tukey’s range tests (*p* < 0.05).

To test the *in vivo* function of *AT1G72910*, *AT1G72940*, and *ADR1-LIKE 2*, we used artificial miRNAs (amiRNAs) to decrease their expression. We designed amiRNAs that target all three NLR genes (Figure 4a) and expressed them in *upf1/3* mutants under the control of the 35S promoter (*35S::amiR-NLRs upf1/3*). First we confirmed that mRNA levels of *AT1G72910*, *AT1G72940*, and *ADR1-LIKE 2* were decreased in the *35S::amiR-NLRs upf1/3* plants (Figure 4b). We found that *35S::amiR-NLRs upf1/3* mutants did not show the severe growth arrest (Figure 4c), indicating that knocking down *AT1G72910*, *AT1G72940*, and *ADR1-LIKE 2* together partially rescued the *upf1/3* phenotype. Consistent with the phenotypic rescue, *PR1* transcript levels were significantly lower in *35S::amiR-NLRs upf1/3* mutants than in *upf1/3* mutants (Figure 4d), suggesting that the spontaneous elicitation of defense response was diminished in *35S::amiR-NLRs upf1/3* plants. Despite the phenotypic rescue, PTC+ transcripts of *AT2G45670* accumulated to similar levels in *upf1/3* mutants and *35S::amiR-NLRs upf1/3* plants (Figure 4e), suggesting that a similar level of impairment of NMD still occurred in both plants.

### 2.5. Overexpression of AT1G72910, AT1G72940, and ADR1-LIKE 2 Results in Autoimmunity in WT Plants

**Figure 5:**
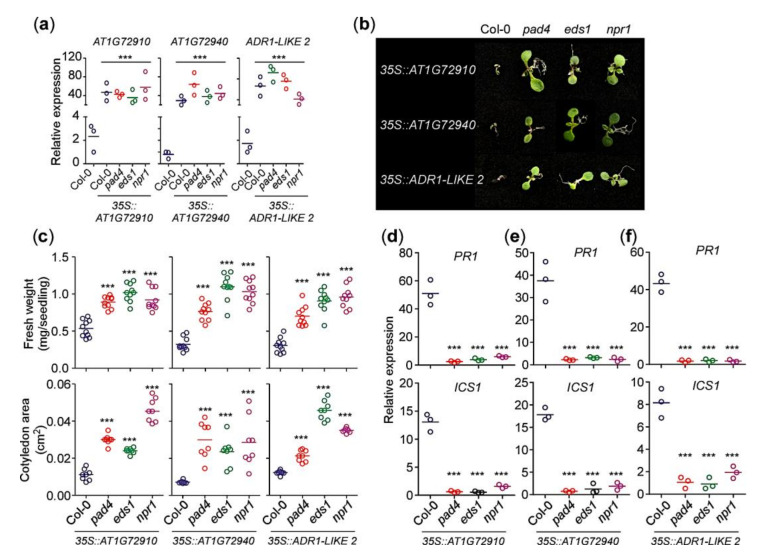
Overexpression of *AT1G72910*, *AT1G72940*, and *ADR1-LIKE 2* results in autoimmunity in the WT background. (**a**) Validation of overexpression of *AT1G72910*, *AT1G72940*, and *ADR1-LIKE 2* in transgenic lines in T_1_ lines at 16 °C. (**b**) Induction of a growth arrest phenotype by overexpression of *AT1G72910, AT1G72940*, and *ADR1-LIKE 2* in Col-0 plants at 16 °C. Note that the growth arrest phenotype was not seen in the *pad4*, *eds*, and *npr1* backgrounds, although *AT1G72910*, *AT1G72940*, and *ADR1-LIKE 2* were overexpressed. (**c**) Quantification of fresh weight (mg per seedling) and cotyledon area (cm^2^) of the transgenic seedlings overexpressing *AT1G72910*, *AT1G72940*, or *ADR2-LIKE 2* at 16 °C in the Col-0, *pad4*, *eds*, and *npr1* backgrounds. (**d**–**f**) *PR1* and *ICS1* expression in transgenic seedlings overexpressing *AT1G72910* (**d**), *AT1G72940* (**e**) or *ADR2-LIKE 2* (**f**) at 16 °C in the Col-0, *pad4*, *eds*, and *npr1* backgrounds. All of the experiments were conducted at 16 °C under long day conditions. Letters indicate significant difference from one-way ANOVA followed by Tukey’s range tests (*p* < 0.05) ***: *p* ≤ 0.001.

We showed that *AT1G72910*, *AT1G72940*, and *ADR1-LIKE 2* were upregulated in NMD-deficient plants (Figure 2e), and mutations in these genes resulted in increased susceptibility to bacterial pathogens (Figure 3e). Therefore, we expected overexpression of these genes to cause a strong elicitation of defense response. To confirm this hypothesis, we analyzed the effect of overexpression of *AT1G72910*, *AT1G72940*, and *ADR1-LIKE 2* in the wild-type background. For comparison, we introduced the overexpression cassette of each NLR gene into *pad4*, *eds1*, and *npr1* mutants. We first confirmed overexpression of each NLR gene in all genotypes via qPCR (Figure 5a). Overexpression of each of the NLR genes in the wild-type Col-0 background resulted in severely stunted growth (Figure 5b), characterized by low fresh weight and small cotyledons (Figure 5c). However, overexpression of *AT1G72910*, *AT1G72940*, and *ADR1-LIKE 2* in the *pad4* and *eds1* mutant background did not induce growth defects (Figure 5b), as shown by the significantly higher fresh weights and larger cotyledons (Figure 5c). These results indicated that overexpression of *AT1G72910*, *AT1G72940*, and *ADR1-LIKE 2* was unable to induce defense responses in the absence of functional *PAD4* and *EDS1*. Overexpressing *AT1G72910*, *AT1G72940*, and *ADR1-LIKE 2* in *npr1* mutants also failed to produce growth defects (Figure 5b,c). Consistent with the severe growth arrest, mRNA levels of defense marker gene *PR1* and SA biosynthesis gene *ICS1* increased in WT plants (Figure 5d-f). Taken together, our results suggest that *AT1G72910*, *AT1G72940*, and *ADR1-LIKE2* play an important role in eliciting constitutive immune responses in *upf1/3* mutant plants at 16 °C in a *PAD4/EDS1*- and SA-signaling-dependent manner.

## 3. Discussion

The NMD mRNA surveillance system prevents translation from aberrant transcripts. NMD is indispensable for the proper growth and development of eukaryotic organisms as its deficiency leads to a number of phenotypic defects in animals and plants [9,41]. In plants, NMD regulates a number of processes including immune response, cell differentiation, and circadian rhythms [27,42,43]. Genome-wide studies revealed that NMD deficiency affects up to 21% of the Arabidopsis transcriptome [6,7,24,27], indicating that NMD is a global phenomenon. GO term analyses of upregulated genes in *upf1/3* mutants revealed significant enrichment of GO terms related to SA biosynthesis/signaling and plant defense (Figure 1e, Figure S1), which is consistent with the previous report [27]. NMD-deficient mutants accumulate higher levels of SA compared to WT plants [8,27]; we found a temperature-dependent increase in the SA levels increased in *upf1/3* mutants (Figure 1g), leading to a strong defense response, consistent with the observed dramatic increase in *PR1* transcript levels (Figure 1h). Higher accumulation of SA has been linked with the upregulation of *ICS1* [8,27]; however, our results suggest that not only ICS but also the PAL pathway genes contribute to the higher accumulation of SA (Supplementary Figure S2a), which is further supported by the partial phenotypic rescue of ABT-treated *upf1/3* mutants at 16 °C (Supplementary Figure S2b).

Temperature is a key environmental factor that influences growth and development of plants. It is known to modulate the activation of the defense pathway and ultimately affects the balance between immune responsiveness and growth in Arabidopsis [22]. AS is a typical example that is affected by temperature. In Arabidopsis, a number of studies have shown the impact of temperature on AS [23,44]. For instance, AS of circadian clock genes and NLR genes is sensitive to temperature. Some of these AS events trigger NMD and hence contribute directly to the stability and abundance of transcripts in response to temperature changes [23,44,45]. Temperature is also known to modulate the growth arrest phenotype of NMD-deficient mutants, such that at lower temperatures, the NMD-deficient mutants exhibit a severe growth-arrest phenotype, whereas at higher ambient temperatures they are almost indistinguishable from WT plants [9,23].

Plant responses to invading pathogens are modulated by a complex network of signaling cascades. In PTI, NLRs sense effectors from the pathogen and induce downstream defense components through the EDS1–PAD4 complex. EDS1 and PAD4 are required for SA accumulation [31,32] and SA induces EDS1 and PAD4 expression, thus amplifying the defense signal via a positive feedback loop [36,37,38]. NLRs are usually activated upon pathogen effector recognition to trigger innate immune responses. Their transcript levels must be tightly regulated to avoid autoimmunity, for instance, upregulation of the *SNC1* gene results in stunted growth caused by the constitutive defense elicitation [46]. Most of the NLR transcripts contain NMD signatures that trigger NMD machinery resulting in transcript degradation or translational suppression, to ensure the proper growth and development under normal conditions [19,28,47]. A recent study reported over 95% of the TNLs and 76% of the coiled-coil-(CC)-containing NLRs (CNLs) were the potential NMD substrates [24]. However, many NLR genes have not been functionally characterized. In this study, we identified and characterized three NLR genes that are post-transcriptionally regulated by NMD in a temperature-dependent manner to suppress autoimmunity and to keep the proper balance between growth and defense. *AT1G72910*, *AT1G72940*, and *ADR1-LIKE 2* were upregulated only at a low temperature in *upf1/3* mutants (Figure 2). Consistent with our idea that *AT1G72910*, *AT1G72940*, and *ADR1-LIKE 2* mediate the low-temperature-specific growth arrest in NMD-impaired mutants, their T-DNA mutants showed higher susceptibility to a bacterial pathogen and increased *PR1* mRNA levels. Furthermore, knocking down these three NLR genes in the *upf1/3* mutant background rescued the stunted growth of *upf1/3* mutants at 16 °C (Figure 4), suggesting that these NLRs are important mediators of the severe developmental defects at lower temperatures.

Overexpression of *AT1G72910*, *AT1G72940*, and *ADR1-LIKE 2* caused stunted growth at 16 °C, and the phenotype required *PAD4*, *EDS1*, and *NPR1* (Figure 5). Similarly, transgenic plants overexpressing *RPS4*, a well-studied NLR, in the Col-0 background conferred resistance against bacterial pathogen *Pst DC3000* in an *EDS1*-dependent manner, as the *RPS4* overexpression was unable to confer resistance in the absence of *EDS1* [48]. Impaired NMD leads to activation of ETI in Arabidopsis [9] and this process relies on NLR proteins. As the expression of NLRs must be tightly regulated at multiple levels to prevent the inappropriate activation of immune responses for the proper balance between growth and immunity [21], it would be interesting to further investigate their temperature-dependent expression patterns and their molecular mechanisms in low-temperature-specific triggering of plant immunity in NMD-impaired mutants.

In conclusion, our findings highlight the functional importance of three uncharacterized NLR genes in the temperature-conditioned plant immunity, such that *AT1G72910*, *AT1G72940*, and *ADR1-LIKE 2* genes are involved in autoimmunity under the NMD-compromised conditions. Further studies on NMD-mediated temperature-dependent regulation of *AT1G72910*, *AT1G72940*, and *ADR1-LIKE 2* genes and the downstream components should increase our understanding of the mechanisms underlying the maintenance of the proper balance between growth and defense responses to optimize plant fitness.

## 4. Materials and Methods

### 4.1. Plant Materials and Growth Conditions

The Arabidopsis thaliana *upf1-5* (SALK_112922), *upf3-1* (SALK_025175), and *upf1-5 upf3-1* mutants were previously described [25,26,49]. For phenotyping, RNA-seq analyses, and quantitative reverse-transcription PCR (qPCR) analyses at different temperatures, 7-day-old seedlings (27 °C), 8-day-old seedlings (23 °C), and 12-day-old seedlings (16 °C) grown under standard long day (LD) conditions (16:8 h light:dark) were used, as these plants were at identical developmental stages [50]. For dark-grown plants, seeds were germinated on half strength Murashige and Skoog (MS) plates covered with aluminum foil. 1-Aminobenzotriazole (Sigma) was used to inhibit SA biosynthesis.

### 4.2. Inhibition of Translation by Cycloheximide (CHX)

To inhibit protein synthesis using CHX, a potent NMD inhibitor, wild-type plants grown at 23 °C for 6 to 7 days were vacuum infiltrated with 20 µM CHX and transferred to 16 °C, 23 °C, and 27 °C. Samples were harvested 5 h after the temperature shift and analyzed [49].

### 4.3. Analyses of Cell Death and Pathogen Assays

Rosette leaves from ~3- to 4-week-old plants were submerged in trypan blue solution (Sigma-Aldrich, Missouri, USA) for 3 h [51]. To detect superoxide ion (O_2_^−^) and hydrogen peroxide (H_2_O_2_), 1 mg/mL nitro blue tetrazolium (NBT) (pH 7.5), and 1 mg/mL 3,3′-diaminobenzidine (DAB) (pH 5.5) [52] were used, respectively. Pathogen challenge experiments were performed according to the previously published procedures [53]. All genotypes were groswn at 23 °C under short day conditions for 4 weeks. They were processed for pathogen assays using the *Pseudomonas syringae pv.* tomato bacterial strain DC3000 (*Pst* DC3000) and were then incubated at 16 °C and 27 °C.

### 4.4. RNA Sequencing

Approximately 100 seedlings were harvested at Zeitgeber time 16 (ZT16) and pooled for RNA extraction using Plant RNA Purification Reagent (Invitrogen). For RNA-seq, an Illumina TruSeq Stranded Total RNA Sample Prep kit was used for library preparation according to the manufacturer’s instructions. The libraries were sequenced with an Illumina HiSeq 2000 sequencer. The raw sequencing data are available at NCBI under accession number GSE134538.

### 4.5. Public Transcriptome Data and Bioinformatics Analyses

A list of public datasets that were used in this study is given in Supplementary Table S2. Datasets from wild-type seedlings treated with flg22 (GSE51720, GSE146189 and GSE99936) [54,55,56], exogenous SA (SRP031882 and GSE125378) [57], and CHX (GSE41432) [6] were used in addition to the RNA-seq data for *upf1*, *upf3*, and *upf1 upf3* (*upf1/3*) mutants at 23 °C (GSE87851) [26]. The Fastq files of RNA-seq data generated in this study (GSE134538) as well as from public datasets, were checked for quality with FastQC (http://www.bioinformatics.babraham.ac.uk/projects/fastqc). The good-quality RNA-seq datasets were subjected to adapter trimming using Sickle (https://github.com/najoshi/sickle) and mapped against the Arabidopsis reference genome TAIR10 using TopHat 2 (https://ccb.jhu.edu/software/tophat/index.shtml) with the optional parameter “—b2-very-sensitive”. The mapped reads were assembled and normalized transcript levels were determined using Cufflinks version 2.2.1 [58] with the optional parameters “—verbose” and “—no-update-check”. The cufflinks option “—library-type” was set according to the dataset used. For coverage maps, the aligned data in bam format were indexed and visualized using Integrated Genome Browser (IGB) (http://bioviz.org/). Cuffdiff 2 was used to identify differentially expressed genes (DEGs) [58] with the parameters “—min-reps-for-js-test” set as 1, and the “—library-type” parameter was set according to the dataset used. The output of Cuffdiff was further analyzed and visualized using the R package CummeRbund [59]. Heatmaps were generated using Papillon (https://github.com/domenico-somma/Papillon). DEGs were defined as genes with fold-change (FC) values of two or more and fragments per kilobase of transcript per million (FPKM) values of two or more. GO analysis was performed with BINGO [60] and PLAZA [61].

### 4.6. qPCR Expression Analyses

qPCR was used to validate the RNA-seq data obtained from the *upf* mutants. Total RNA was extracted from seedlings at the identical developmental stage at ZT16 at different temperatures. Plant RNA purification reagent (Invitrogen) was used for RNA extraction. The DNAse I treated RNA (~2 µg) was reverse transcribed into cDNA using MMLV enzyme (ELPIS Biotech). Quantitative expression analysis was performed using 2X A-Star Real Time PCR Master Mix (BioFACT). Two reference genes, *PP2AA3* (*AT1G13320*) and a *SAND* family gene (*AT2G28390*), were used to normalize the data [62]. All qPCR experiments were conducted in three biological replicates, each with three technical replicates. The statistical significance of differences in gene expression levels among samples was assessed using one-way ANOVA with a 0.05 level of significance (95% confidence interval). Details about the primers used in this study are shown in Supplementary Table S3.

### 4.7. Quantification of Phytohormones and Chlorophyll Contents

To determine endogenous phytohormone levels of the *upf* mutants at different temperatures, gas chromatography (GC) with a mass spectrometry detector (Agilent GC 6890N/MS 5973N) was performed in SIM mode. The calculations were done based on selected internal standards (d4-SA, d7-PAA, d6-ABA, d5-IAA, dihydroxy-JA, d2-GA1, d2-GA4, and d2-GA20). The phytohormones were isolated from 300 mg whole-plant samples from plants grown on MS medium for 3 weeks [63]. Prior to GC analysis, the samples were methylated and trimethylsilylated as described [64]. All measurements were done with three biological replicates. Chlorophyll quantification was performed by measuring absorbance at 665, 649, and 470 nm as described [65].

### 4.8. Determination of mRNA Degradation Kinetics

Cordycepin (150 µg/mL) was used to inhibit transcription in 3-week-old wild-type and *upf1/3* mutants [33]. Leaves were harvested after 0, 1, 2, 3, and 4 h of treatment, and the transcript levels of the genes of interest were determined via qPCR. The half-lives of the mRNAs were calculated by nonlinear least-square regression using GraphPad Prism 7.0 as described previously [66]. Briefly, the mean Ct values for each time point was normalized to the mean Ct value of *t* = 0, to get the normalized Ct value. Relative abundance for each time point was then calculated as 2 to the power of normalized Ct value. mRNA decay rate was determined for the relative abundances using non-linear regression curve fitting in GraphPad Prism.

## Supplementary Materials

Supplementary materials can be found at https://www.mdpi.com/1422-0067/21/21/7986/s1. Figure S1. RNA-seq analysis of *upf* mutants at different temperatures. Figure S2. Involvement of SA and TLN immunity in the severe stunted phenotype of *upf1/3* mutants at 16 °C. Figure S3. Expression profiles and clustering of the selected NLR genes upon CHX treatment at different temperatures. Table S1. List and fold-change of NLR genes, upregulated in *upf* mutants compared to WT plants, at 16 °C. Table S2. List of public transcriptome datasets that were used in this study. Table S3. List of primers used in this study.

## Abbreviations

NLR	Nucleotide-binding, Leucine-rich Repeat
SA	Salicylic acid
CHX	Cycloheximide
DEGs	Differentially expressed genes
HR	Hypersensitive response

## References

[1] Hua J. (2013). Modulation of plant immunity by light, circadian rhythm, and temperature. Curr. Opin. Plant Biol..

[2] Huot B., Castroverde C.D.M., Velásquez A.C., Hubbard E., Pulman J.A., Yao J., Childs K.L., Tsuda K., Montgomery B.L., He S.Y. (2017). Dual impact of elevated temperature on plant defence and bacterial virulence in Arabidopsis. Nat. Commun..

[3] Gangappa S.N., Berriri S., Kumar S.V. (2017). PIF4 Coordinates Thermosensory Growth and Immunity in Arabidopsis. Curr. Biol..

[4] Schweingruber C., Rufener S.C., Zünd D., Yamashita A., Mühlemann O. (2013). Nonsense-mediated mRNA decay — Mechanisms of substrate mRNA recognition and degradation in mammalian cells. Biochim. Biophys. Acta (BBA) Bioenergy.

[5] Isken O., Maquat L.E. (2008). The multiple lives of NMD factors: Balancing roles in gene and genome regulation. Nat. Rev. Genet..

[6] Drechsel G., Kahles A., Kesarwani A.K., Stauffer E., Behr J., Drewe P., Rätsch G., Wachter A. (2013). Nonsense-Mediated Decay of Alternative Precursor mRNA Splicing Variants Is a Major Determinant of the Arabidopsis Steady State Transcriptome. Plant Cell.

[7] Kurihara Y., Matsui A., Hanada K., Kawashima M., Ishida J., Morosawa T., Tanaka M., Kaminuma E., Mochizuki Y., Matsushima A. (2009). Genome-wide suppression of aberrant mRNA-like noncoding RNAs by NMD in Arabidopsis. Proc. Natl. Acad. Sci. USA.

[8] Jeong H.-J., Kim Y.J., Kim S.H., Kim Y.-H., Lee I.-J., Shin J.S. (2011). Nonsense-Mediated mRNA Decay Factors, UPF1 and UPF3, Contribute to Plant Defense. Plant Cell Physiol..

[9] Riehs-Kearnan N., Gloggnitzer J., Dekrout B., Jonak C., Riha K. (2012). Aberrant growth and lethality of Arabidopsis deficient in nonsense-mediated RNA decay factors is caused by autoimmune-like response. Nucleic Acids Res..

[10] Bari R., Jones J.D.G. (2008). Role of plant hormones in plant defence responses. Plant Mol. Biol..

[11] Pieterse C.M., Van Der Does D., Zamioudis C., Leon-Reyes A., Van Wees S.C. (2012). Hormonal Modulation of Plant Immunity. Annu. Rev. Cell Dev. Biol..

[12] Wildermuth M.C., Dewdney J., Wu G., Ausubel F.M. (2001). Isochorismate synthase is required to synthesize salicylic acid for plant defence. Nat. Cell Biol..

[13] Chen Z., Zheng Z., Huang J., Lai Z., Fan B. (2009). Biosynthesis of salicylic acid in plants. Plant Signal. Behav..

[14] Lee H.-J., Park Y.-J., Seo P.J., Kim J.-H., Sim H.-J., Kim S.-G., Park C.-M. (2015). Systemic Immunity Requires SnRK2.8-Mediated Nuclear Import of NPR1 in Arabidopsis. Plant Cell.

[15] Tada Y., Spoel S.H., Pajerowska-Mukhtar K., Mou Z., Song J., Wang C., Zuo J., Dong X. (2008). Plant Immunity Requires Conformational Charges of NPR1 via S-Nitrosylation and Thioredoxins. Science.

[16] Jones J.D.G., Dangl J.L. (2006). The plant immune system. Nat. Cell Biol..

[17] Fu Z.Q., Dong X. (2013). Systemic Acquired Resistance: Turning Local Infection into Global Defense. Annu. Rev. Plant Biol..

[18] Dodds P.N., Rathjen J.P. (2010). Plant immunity: Towards an integrated view of plant–pathogen interactions. Nat. Rev. Genet..

[19] Maekawa T., A Kufer T., Schulze-Lefert P. (2011). NLR functions in plant and animal immune systems: so far and yet so close. Nat. Immunol..

[20] Bernoux M., Burdett H., Williams S.J., Zhang X., Chen C., Newell K., Lawrence G.J., Kobe B., Ellis J.G., Anderson P.A. (2016). Comparative Analysis of the Flax Immune Receptors L6 and L7 Suggests an Equilibrium-Based Switch Activation Model. Plant Cell.

[21] Staiger D., Korneli C., Lummer M., Navarro L. (2012). Emerging role for RNA-based regulation in plant immunity. N. Phytol..

[22] Alcázar R., Parker J.E. (2011). The impact of temperature on balancing immune responsiveness and growth in Arabidopsis. Trends Plant Sci..

[23] Gloggnitzer J., Akimcheva S., Srinivasan A., Kusenda B., Riehs N., Stampfl H., Bautor J., Dekrout B., Jonak C., Jiménez-Gómez J.M. (2014). Nonsense-Mediated mRNA Decay Modulates Immune Receptor Levels to Regulate Plant Antibacterial Defense. Cell Host Microbe.

[24] Jung H.W., Panigrahi G.K., Jung G.Y., Lee Y.J., Shin K.H., Sahoo A., Choi E.S., Lee E., Kim K.M., Yang S.H. (2020). Pathogen-Associated Molecular Pattern-Triggered Immunity Involves Proteolytic Degradation of Core Nonsense-Mediated mRNA Decay Factors During the Early Defense Response. Plant Cell.

[25] Arciga-Reyes L., Wootton L., Kieffer M., Davies B. (2006). UPF1 is required for nonsense-mediated mRNA decay (NMD) and RNAi in Arabidopsis. Plant J..

[26] Nasim Z., Fahim M., Ahn J.H. (2017). Possible Role of MADS AFFECTING FLOWERING 3 and B-BOX DOMAIN PROTEIN 19 in Flowering Time Regulation of Arabidopsis Mutants with Defects in Nonsense-Mediated mRNA Decay. Front. Plant Sci..

[27] Rayson S., Arciga-Reyes L., Wootton L., Zabala M.D.T., Truman W., Graham N., Grant M., Davies B. (2012). A Role for Nonsense-Mediated mRNA Decay in Plants: Pathogen Responses Are Induced in Arabidopsis thaliana NMD Mutants. PLoS ONE.

[28] Raxwal V.K., Simpson C.G., Gloggnitzer J., Entinze J.C., Guo W., Zhang R., Brown J.W., Riha K. (2020). Nonsense-mediated RNA Decay Factor UPF1 is Critical for Post-transcriptional and Post-translational Gene Regulation in Arabidopsis. Plant Cell.

[29] Mammarella N.D., Cheng Z., Fu Z.Q., Daudi A., Bolwell G.P., Dong X., Ausubel F.M. (2015). Apoplastic peroxidases are required for salicylic acid-mediated defense against Pseudomonas syringae. Phytochemistry.

[30] Neuenschwander U., Vernooij B., Friedrich L., Uknes S., Kessmann H., Ryals J. (1995). Is hydrogen peroxide a second messenger of salicylic acid in systemic acquired resistance?. Plant J..

[31] Feys B.J., Moisan L.J., Newman M., Parker J.E. (2001). Direct interaction between the Arabidopsis disease resistance signaling proteins, EDS1 and PAD4. EMBO J..

[32] Rustérucci C., Aviv D.H., Holt B.F., Dangl J.L., Parker J.E. (2001). The disease resistance signaling components EDS1 and PAD4 are essential regulators of the cell death pathway controlled by LSD1 in Arabidopsis. Plant Cell.

[33] Johnson M.A., Pérez-Amador M.A., Lidder P., Green P.J. (2000). Mutants of Arabidopsis defective in a sequence-specific mRNA degradation pathway. Proc. Natl. Acad. Sci. USA.

[34] Xia S., Cheng Y.T., Huang S., Win J., Soards A., Jinn T.-L., Jones J.D., Kamoun S., Chen S., Zhang Y. (2013). Regulation of Transcription of Nucleotide-Binding Leucine-Rich Repeat-Encoding Genes SNC1 and RPP4 via H3K4 Trimethylation. Plant Physiol..

[35] Van Loon L.C. (2016). The Intelligent Behavior of Plants. Trends Plant Sci..

[36] Cui H., Tsuda K., Parker J.E. (2015). Effector-Triggered Immunity: From Pathogen Perception to Robust Defense. Annu. Rev. Plant Biol..

[37] Sano S., Aoyama M., Nakai K., Shimotani K., Yamasaki K., Sato M.H., Tojo D., Suwastika I.N., Nomura H., Eshiina T. (2014). Light-dependent expression of flg22-induced defense genes in Arabidopsis. Front. Plant Sci..

[38] Zhou N., Tootle T.L., Tsui F., Klessig D.F., Glazebrook J. (1998). PAD4 Functions Upstream from Salicylic Acid to Control Defense Responses in Arabidopsis. Plant Cell.

[39] Roberts M.R., Paul N.D. (2006). Seduced by the dark side: Integrating molecular and ecological perspectives on the influence of light on plant defence against pests and pathogens. N. Phytol..

[40] Pink B., Mueller M.J., Berger S. (2004). Light conditions influence specific defence responses in incompatible plant?pathogen interactions: Uncoupling systemic resistance from salicylic acid and PR-1 accumulation. Planta.

[41] Wittkopp N., Huntzinger E., Weiler C., Saulière J., Schmidt S., Sonawane M., Izaurralde E. (2009). Nonsense-Mediated mRNA Decay Effectors Are Essential for Zebrafish Embryonic Development and Survival. Mol. Cell. Biol..

[42] Bruno I.G., Karam R., Huang L., Bhardwaj A., Lou C.H., Shum E.Y., Song H.-W., Corbett M.A., Gifford W.D., Gecz J. (2011). Identification of a MicroRNA that Activates Gene Expression by Repressing Nonsense-Mediated RNA Decay. Mol. Cell.

[43] Filichkin S.A., Cumbie J.S., Dharmawardhana P., Jaiswal P., Chang J.H., Palusa S.G., Reddy A., Megraw M., Mockler T.C. (2015). Environmental Stresses Modulate Abundance and Timing of Alternatively Spliced Circadian Transcripts in Arabidopsis. Mol. Plant.

[44] Aarts N., Metz M., Holub E., Staskawicz B.J., Daniels M.J., Parker J.E. (1998). Different requirements for EDS1 and NDR1 by disease resistance genes define at least two R gene-mediated signaling pathways in Arabidopsis. Proc. Natl. Acad. Sci. USA.

[45] Gassmann W. (2008). Alternative Splicing in Plant Defense. The Future of HIV-1 Therapeutics.

[46] Kim S.H., Gao F., Bhattacharjee S., Adiasor J.A., Nam J.C., Gassmann W. (2010). The Arabidopsis Resistance-Like Gene SNC1 Is Activated by Mutations in SRFR1 and Contributes to Resistance to the Bacterial Effector AvrRps4. PLoS Pathog..

[47] Karasov T.L., Chae E., Herman J.J., Bergelson J. (2017). Mechanisms to Mitigate the Trade-Off between Growth and Defense. Plant Cell.

[48] Heidrich K., Tsuda K., Blanvillain-Baufumé S., Wirthmueller L., Bautor J., Parker J.E. (2013). Arabidopsis TNL-WRKY domain receptor RRS1 contributes to temperature-conditioned RPS4 auto-immunity. Front. Plant Sci..

[49] Ehori K., Watanabe Y. (2005). UPF3 suppresses aberrant spliced mRNA in Arabidopsis. Plant J..

[50] Boyes D.C., Zayed A.M., Ascenzi R., McCaskill A.J., Hoffman N.E., Davis K.R., Görlach J. (2001). Growth Stage–Based Phenotypic Analysis of Arabidopsis. Plant Cell.

[51] Van Wees S.C. (2008). Phenotypic Analysis of Arabidopsis Mutants: Trypan Blue Stain for Fungi, Oomycetes, and Dead Plant Cells. Cold Spring Harb. Protoc..

[52] Thordal-Christensen H., Zhang Z., Wei Y., Collinge D.B. (1997). Subcellular localization of H2O2 in plants. H2O2 accumulation in papillae and hypersensitive response during the barley-powdery mildew interaction. Plant J..

[53] Liu X., Sun Y., Kørner C.J., Du X., Vollmer M.E., Pajerowska-Mukhtar K.M. (2015). Bacterial Leaf Infiltration Assay for Fine Characterization of Plant Defense Responses using the Arabidopsis thaliana-Pseudomonas syringae Pathosystem. J. Vis. Exp..

[54] Rallapalli G., Kemen E.M., Robert-Seilaniantz A., Segonzac C., Etherington G.J., Sohn K.H., MacLean D., Jones J.D.G. (2014). EXPRSS: An Illumina based high-throughput expression-profiling method to reveal transcriptional dynamics. BMC Genom..

[55] Bazin J., Mariappan K., Jiang Y., Blein T., Voelz R., Crespi M., Hirt H. (2020). Role of MPK4 in pathogen-associated molecular pattern-triggered alternative splicing in Arabidopsis. PLoS Pathog..

[56] Latrasse D., Jégu T., Li H., Zelicourt A.D.J.D., Raynaud C., Legras S., Gust A., Samajova O., Veluchamy A., Rayapuram N. (2017). MAPK-triggered chromatin reprogramming by histone deacetylase in plant innate immunity. Genome Biol..

[57] Caarls L., Van Der Does D., Hickman R., Jansen W., Van Verk M.C., Silvia P., Lorenzo O., Solano R., Pieterse C.M.J., Van Wees S.C. (2016). Assessing the Role of ETHYLENE RESPONSE FACTOR Transcriptional Repressors in Salicylic Acid-Mediated Suppression of Jasmonic Acid-Responsive Genes. Plant Cell Physiol..

[58] Trapnell C., Roberts A., A Goff L., Pertea G., Kim D., Kelley D.R., Pimentel H., Salzberg S.L., Rinn J.L., Pachter L. (2012). Differential gene and transcript expression analysis of RNA-seq experiments with TopHat and Cufflinks. Nat. Protoc..

[59] Goff L.A., Trapnell C., Kelley D. (2012). CummeRbund: Visualization and exploration of Cufflinks high-throughput sequencing data. R Package Vers..

[60] Maere S., Heymans K., Kuiper M. (2005). BiNGO: A Cytoscape plugin to assess overrepresentation of Gene Ontology categories in Biological Networks. Bioinformatics.

[61] Proost S., Van Bel M., Vaneechoutte D., Van De Peer Y., Inzé D., Mueller-Roeber B., Vandepoele K. (2014). PLAZA 3.0: An access point for plant comparative genomics. Nucleic Acids Res..

[62] Hong S.M., Bahn S.C., Lyu A., Jung H.S., Ahn J.H. (2010). Identification and Testing of Superior Reference Genes for a Starting Pool of Transcript Normalization in Arabidopsis. Plant Cell Physiol..

[63] Nehela Y., Hijaz F., Elzaawely A.A., El-Zahaby H.M., Killiny N. (2016). Phytohormone profiling of the sweet orange (Citrus sinensis (L.) Osbeck) leaves and roots using GC–MS-based method. J. Plant Physiol..

[64] Hijaz F., Killiny N. (2014). Collection and Chemical Composition of Phloem Sap from Citrus sinensis L. Osbeck (Sweet Orange). PLoS ONE.

[65] Frye C.A., Tang D., Innes R.W. (2001). Negative regulation of defense responses in plants by a conserved MAPKK kinase. Proc. Natl. Acad. Sci. USA.

[66] Ratnadiwakara M., Änkö M.-L. (2018). mRNA Stability Assay Using Transcription Inhibition by Actinomycin D in Mouse Pluripotent Stem Cells. Bio-Protocol.

