# Supplementary material for: Role of AT1G72910, AT1G72940, and ADR1-LIKE 2 in Plant Immunity under Nonsense-Mediated mRNA Decay-Compromised Conditions at Low Temperatures
Source: Int J Mol Sci. 2020 Oct 27;21(21):7986. doi: 10.3390/ijms21217986 (PMC7663611; doi:10.3390/ijms21217986)
Supplement: Supplementary file 1 [file ijms-21-07986-s001.pdf]

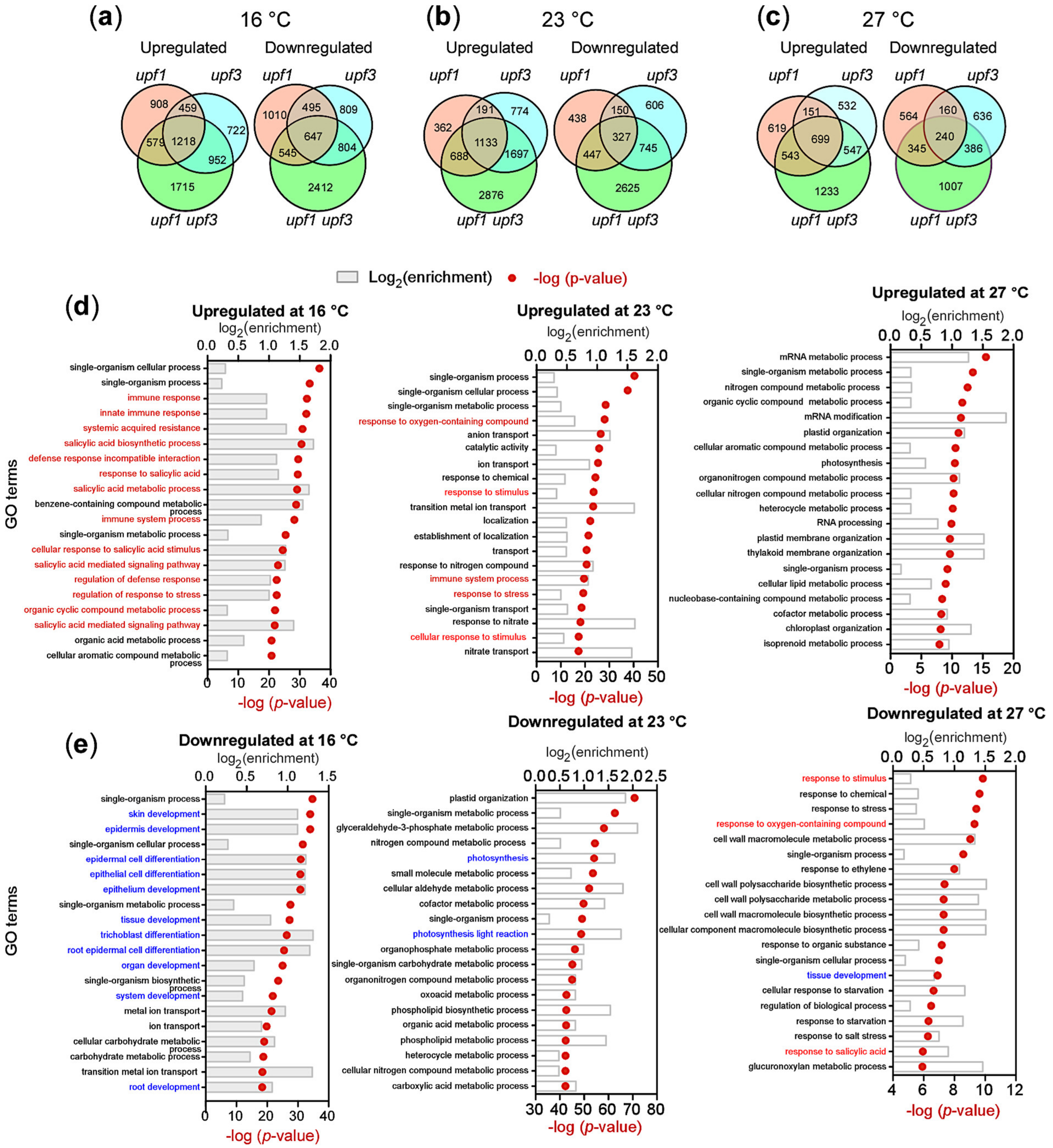

Figure S1. GO analysis of DEGs in the *upf* mutants. (**a**–**c**) Venn diagram showing the DEGs in *upf1*, *upf3*, and *upf1 upf3* plants at 16 °C (**a**), 23 °C (**b**), and 27 °C (**c**). Top 20 GO terms among upregulated (**d**) and downregulated (**e**) genes in *upf1 upf3* at 16, 23 and 27 °C. Y-axis represents GO terms; the primary X-axis represents log_2 enrichment, whereas the secondary X-axis shows the negative log of *p* values. GO terms related to defense are shown in red, whereas GO terms related to growth/development are shown in blue.

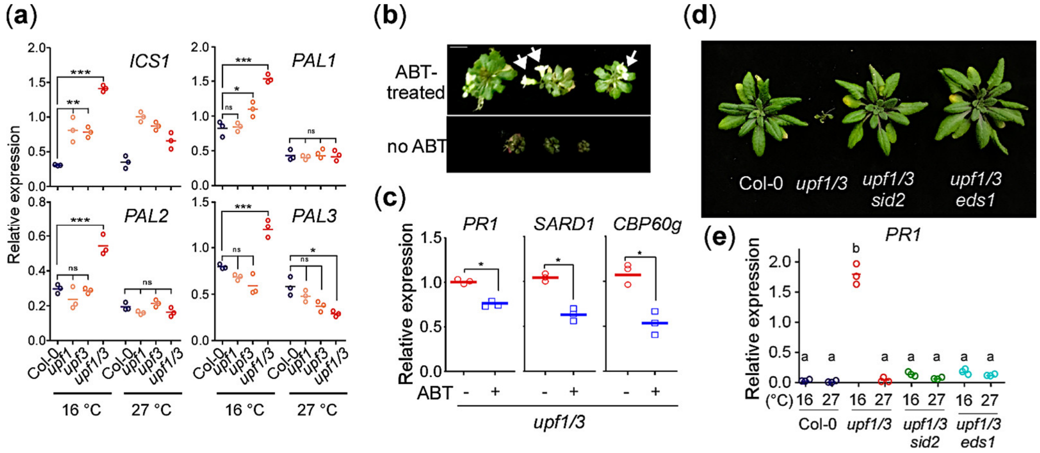

Figure S2. A strong immune response underlies the phenotype of *upf1 upf3* plants 16 °C. (**a**) mRNA levels of *ICS1* and *PAL1-3* in the upf mutants at different temperatures. (**b**) Phenotypic rescue of *upf1 upf3* grown at 16 °C by ABT treatment. Note that normal flowers (arrows) formed in ABT-treated *upf1 upf3* plants. Scale bar = 1 cm. (**c**) Decrease in *PR1*, *SARD1*, and *CBP60g* transcript levels in ABT-treated *upf1 upf3* plants. (**d**) Rescue of the growth arrest phenotype of *upf1/3* mutants by the introduction of *sid2* and *eds1* mutation at 16 °C. (**e**) Increase of *PR1* mRNA levels in *upf1/3* mutants and decrease of *PR1* mRNA levels in *upf1/3 sid2* and *upf1/3 eds1* mutants at 16 °C. Letters indicate significant difference from one-way ANOVA followed by Tukey's range tests, whereas the asterisk signs represents the p-value derived from Student's t-test i.e. ($p <$ 0.05) *: $p < 0.05$; **: $p < 0.01$, ***: $p \leq 0.001$; ns: not significant.

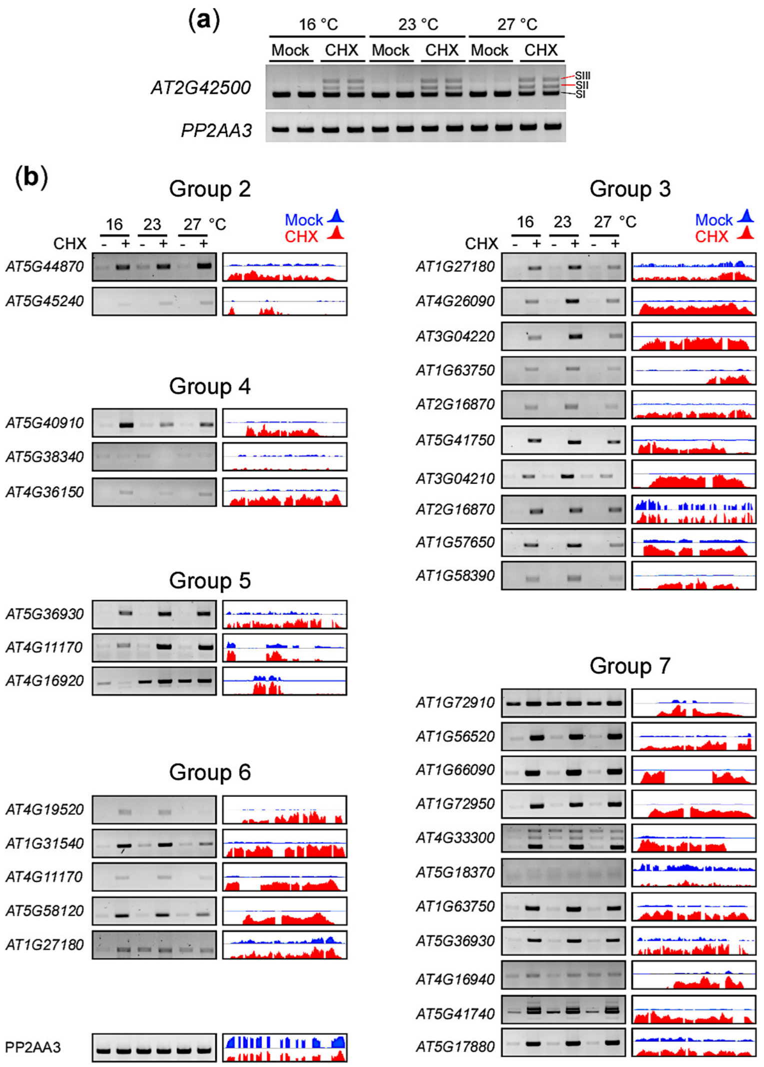

Figure S3. Temperature-dependent regulation of NLR genes in CHX-induced NMD-inhibited WT seedlings. (**a**) Gel picture showing the RT-PCR amplification of *AT2G42500*, a known NMD target, in WT plants under mock- and CHX-treatment conditions at different temperatures. (**b**) The expression levels of 34 NLR genes belonged to groups 2–7 determined by RT-PCR (left), whereas the read coverage graphs from the public transcriptome data (GSE41432) representing the expression of these NLR genes upon CHX-treatment (right). Classification of these NLR genes were based on their expression patterns (Figure 2b). *PP2AA3* was used as a loading control.

Table 1. List and Fold-Change of NLR Genes, Upregulated in Upf Mutants Compared to WT Plants, at 16 °C.

**Temperature**	**16 °C**		**27 °C**	
Gene IDs	Col-0	*upf1/3*	Col-0	*upf1/3*
AT5G58120	2.9	5.3	0.9	2.3
AT1G58390	0.18	1.2	0.1	0.8
AT4G26090	3.7	6.3	1.7	2.1
AT4G16940	0.26	1.5	0.3	0.45
AT5G04720	13.8	24.9	4.3	7.4
AT5G46520	1.3	3.4	0.45	0.4
AT1G66090	1.9	5.8	1.1	1.3
AT1G31540	7.5	18.6	3.4	4.4
AT4G33300	18.9	40.3	12.4	15.8
AT5G46270	4.8	9.7	2.2	3.61
AT3G44400	2.8	5.8	1.1	1.7
AT1G72940	5.04	15.2	3.2	4.7
AT5G45240	0.6	1.4	0.3	0.47
AT4G08450	0.2	1.1	0.3	0.4
AT3G04210	7.1	39.2	3.4	6.3
AT5G41740	2.06	13.3	3.8	5.4
AT1G56520	4.2	8.19	1.2	1.9
AT4G11170	0.39	1.8	0.17	0.201
AT4G36150	2.8	5.6	1.2	1.1
AT5G44870	6.4	11.6	2.7	23.2
AT5G40910	14	30.3	9	14
AT5G66900	11.6	22.1	8.4	11.2
AT5G17880	4.9	11.3	1.1	1.8
AT1G33560	3.7	11.6	5.9	7.4
AT3G04220	0.3	1.2	0.2	0.35
AT4G19520	4.6	8.9	2.1	3
AT2G16870	0.8	2.3	1.1	1.6
AT5G18370	1.2	3.7	0.9	1.5
AT5G36930	6.1	12.4	4	3.5
AT1G27180	1.4	3.2	1	1.4
AT3G46710	2.9	5.2	1.2	1.8
AT5G41550	0.18	1.1	0.28	0.386
AT1G72950	0.7	2.5	0.3	0.76
AT5G41750	1.4	5.6	2.3	3.7
AT5G38340	1.7	3.8	0.985	1.4
AT1G63750	3.8	6.9	2.1	3.6
AT1G57650	0.1	1.3	0	0.3
AT4G16920	0.9	3.5	0.3	0.56

Table S2. List of Public Transcriptome Datasets That Were Used in This Study.

**Accession No**	**Sample**	**Treatment**	**Reference**
GSE51720	Col-0	flg22	[55]
GSE146189	Col-0	flg22	[56]
GSE99936	Col-0	flg22	[57]
SRP075162	Col-0	*Pst DC300*	-
GSE125378	Col-0	SA	-

SRP031882	Col-0	SA	[58]
GSE87851	Col-0, *upf1*, *upf3*, *upf1/3*	-	[26]
GSE41432	Col-0	CHX	[6]

Table S3. List of Primers Used in This Study.

Gene	Orientation	Sequence (5′ to 3′)
ADR1-LIKE2	Sense	GAATCGTTGCGTGAGGCGTT
	Antisense	AGCACCATCGGTTGTCACCA
AT1G72910	Sense	GGCCAGAGGATTTCGTCGGA
	Antisense	TCCACGCCGTAGAAGATGGG
AT1G72940	Sense	CGCCGTCGTTGTTGTCTCTG
	Antisense	GCGACATCTCCGATCTGCCT
AT5G41550	Sense	TCTGCGACTTAGCACGGCTT
	Antisense	ATCGAGGTCAGGGTCATCGC
CBP60g	Sense	AATAACGAGGAGGATGAGAACG
	Antisense	TCAGACACGGTAAGAAACATCG
EDS1	Sense	CTGAGTTAGCCGGTGT
	Antisense	TTTCATGTACGGCCCTG
ICS1	Sense	AGGTCCCGCATACATT
	Antisense	GATAACATGCCGGACTTCCT
ICS2	Sense	TGCAGTGTGAAGGACAAGAC
	Antisense	GAAGAGTCTCTCAGGCGTGT
ICS2	Sense	TGCAGTGTGAAGGACAAGAC
	Antisense	GAAGAGTCTCTCAGGCGTGT
PAD4	Sense	TTAGCCGTTGAAGCTCT
	Antisense	CTTCAAGCTCCACGGTGTAAG
PAL1	Sense	TTGGAGCTTTCGAGGAGGAG
	Antisense	TTCCGTTATCGTAGGCTGCT
PAL2	Sense	GAGCTTAAGGCTGTGCTTCC
	Antisense	TTCGTTCCAAGCTCTTCCCT
PAL3	Sense	GGACAGAGTACGAGAACGGT
	Antisense	CCGAACATCCTCTCCGGTTA
PBS3	Sense	CGTACCGATCGTGTCATATGAAG
	Antisense	CTTCACATGCTTGGTTATAACTTGC
PP2AA3	Sense	GCGGTTGTGGAGAACATGATACG
	Antisense	GAACCAAACACAATTCGTTGCTG
PR1	Sense	GCTCTTGTAGGTGCTCTTGTTCTTCC
	Antisense	AGTCTGCAGTTGCCTCTTAGTTGTTC
RING1	Sense	TGAAGAAGACGAGTCGCTGA
	Antisense	GGGCACGACATAAAGGACAG
SAND family	Sense	TTGATCCACTTGCAGACAAGGC
	Antisense	TACCCTTTGGCACACCTGATTG
SARD1	Sense	CCTCAACCAGCCCTACGTTA
	Antisense	TAGTGGCTCGCAGCATATTG
WRKY15	Sense	AAAGGATCTCCACATCCAAGAG
	Antisense	GCGGCGGAGAGAGAATGAT
WRKY18	Sense	CATACGAAGGGACGCATAAC
	Antisense	CCTTTCGTTTTTCTCCAACG
WRKY25	Sense	ACCTCTTCCGATTTCACA
	Antisense	GTTCCATTAAAGCCTT
WRKY33	Sense	TACGAAGGGAAACACAACCA

	Antisense	AAGGCCCGGTATTAGTGTTG
WRKY38	Sense	CACTTACTTTGGCCACCACAC
	Antisense	GGCTTTCCTTCTCCTGATCCT
WRKY46	Sense	GACATGAAACCGACCAAGTCC
	Antisense	CAGTTTCGGAATTCTCAACAGCA
WRKY51	Sense	ACAAGCATCGTTTCGTCGGA
	Antisense	CAACTCGATGACCCGTCTCC
WRKY54	Sense	GCACTGCTCAGAACCATGTCAA
	Antisense	CAAGTCCTCACCTGTCGAAGA
WRKY60	Sense	ACACCAATCCGTTCGACCTT
	Antisense	CGACTCTGCAACTCCTCCAT
WRKY70	Sense	CATGGATTCCGAAGATCACA
	Antisense	CTGGCCACACCAATGACAA

Primers for RT-PCR.

Gene	Orientation	Sequence (5′ to 3′)
AT1G27180	Sense	AGAGGACTCGGTGGCTTTTG
	Antisense	CACAGAAGTTGGTTCGCGTG
AT1G31540	Sense	AGGATCGCTGTGGTCGTTTT
	Antisense	GCGATGTGATCTTCGATGCC
AT1G57650	Sense	TGGTAAAGAAGTGCCTGCGT
	Antisense	GACAAGACACCAACCCTCGT
AT1G58390	Sense	AATGAGAGTGTCGCGGTACG
	Antisense	CTGCTGGTTCTTCCCACGAT
AT1G63750	Sense	TCGTAGACAGGAGCCTTGGA
	Antisense	AAGGTGTTGGGTTCCTTCCC
AT1G66090	Sense	AACCCTTGCCCCTACTCTCA
	Antisense	CTTGGACGTGTACTCCGGTT
AT1G72910	Sense	ATGACTCGAAGCTGGTGGAC
	Antisense	GAGTGGCGACCCTGAGAAAT
AT1G72940	Sense	CAGGGTCGTCACTTATCGCA
	Antisense	AGGCGAATTGTCGGAAGAGT
AT1G72950	Sense	CGCCATTGAAGGGTCGAGAT
	Antisense	TGCCTCCTCTTGCCCAAATC
AT2G16870	Sense	TTGGGTCTACGTGTGGTTGG
	Antisense	AATGGCTTGTCTGCCCACTT
AT3G04210	Sense	CCCGGTCGTAAACACCAAGT
	Antisense	CCCAAAATCTCCGGTCTGCT
AT3G04220	Sense	GGGCGATTAGAGGGTCCAAG
	Antisense	CCCCAGGAGGACCCCATATT
AT4G11170	Sense	TCAGAGGGGAAGATGTCCGT
	Antisense	CCCGCCTCCAGTTATGTTGT
AT4G16920	Sense	ACGAGGCTCCGTGTACTACT
	Antisense	ATGTCATCCCCATCCGCTTC
AT4G16940	Sense	CGTGGTTGCAAGTCGTTGAG
	Antisense	AGTTCTCGCGCAGCTCTATC
AT4G33300	Sense	GGTTGACATGAGCCTCGACA
	Antisense	TGGACTTCATGGTCCCGTTG
AT4G36150	Sense	AACGGTTTCGTCAGCCATCT
	Antisense	AACGTGTAACCAAAAGCGCC
AT5G04720	Sense	CCAGGCTCAGTTGCGTATGT

	Antisense	TCCCTGAACCACTCATCCCA
AT5G17880	Sense	CAGATAGAAAGGGGCGGCAT
	Antisense	ATCGCACCTCATTCAACGGT
AT5G18370	Sense	GCAGTTCAGGAGAGGCTCAG
	Antisense	CCTTAACCGTGGTAGTGCGT
AT5G36930	Sense	GGACTACGCATCATCGGCTT
	Antisense	GTACTGGCAGGGCAGTCTTT
AT5G38340	Sense	GAAGTGCCGTCGAGGATCAA
	Antisense	AGCACGGAACCTCCAGTTTT
AT5G40910	Sense	AGATTCGCGATGTCTTGGCA
	Antisense	CTCTCACAACCGGTAAGCGT
AT5G41550	Sense	TCTGCGACTTAGCACGGCTT
	Antisense	ATCGAGGTCAGGGTCATCGC
AT5G41740	Sense	GCTGAGGGTGACAGTAGCAG
	Antisense	CCCAAAGAATTCGCGACGAC
AT5G41750	Sense	TGTGTCGTGGGTGCATCTTT
	Antisense	AGCCCCCTTACCTACCGAAA
AT5G44870	Sense	TGGTGGTCGAGTTGAGATGC
	Antisense	ACCTTCGCCTTCCTTATGGC
AT5G45240	Sense	AGAAACCAAGCAACCCGACA
	Antisense	ATTGTTGCCTCACGTCCGAT
AT5G46520	Sense	CCGATGCTGTCTCCCACTTT
	Antisense	TCGCGGCAACCTTTCATAGT
AT5G58120	Sense	TAGGTCTCCGTGTCATGGGT
	Antisense	ATTCTCCGTTTCGTGGGCTC
EDS1-PTC+	Sense	ACTTTCCGACCAAATGTGAATG
	Antisense	GTTGACTCTTTGGCTATTGGAGAC
PP2AA3	Sense	AGCCATTGTAGAACTTGCTG
	Antisense	CTATATGCTGCATTGCCCAT
SARD1-PTC+	Sense	GGAAGATCGGAACCGTCCATTTG
	Antisense	ATCCAAAGAAGTCCGAAGTCTG
WRKY38-PTC+	Sense	AAATTCTGATCTCCCTATCCGTTC
	Antisense	ATTGGATGGTTGAGCCGCCTCTTC

Primers for 35S::NLRs Cloning.

Gene	Orientation	Sequence (5′ to 3′)
ADR1-LIKE2	Sense	gacgataaaagatcttctaga ATGGCAGATATAATCGGCGGCG
	Antisense	catattaatgtcgacggtacc CTAATCGTCGAGCCAATCCCTG
AT1G72910	Sense	gacgataaaagatcttctaga ATGTCTTCTCATACTGCAACTA
	Antisense	catattaatgtcgacggtacc TTAAACTAAATCTGTCACTTTC
AT1G72940	Sense	gacgataaaagatcttctaga ATGACTTCTCCTACTGCGACTAAG
	Antisense	catattaatgtcgacggtacc CTAACCAGATCTACCACTTAGA

Primers for 35S::amiR-NLRs Cloning.

Gene	Primer	Sequence (5′ to 3′)
ADR1-LIKE2	I miR-s	gaTAAATCCCCATATATGGGCTCtctctcttttgtattcc
	II miR-a	gaGAGCCCATATATGGGGATTTAtcaaagagaatcaatga
	III miR*s	gaGAACCCATATATGCGGATTTTtcacaggtcgtgatatg
	IV miR*a	gaAAAATCCGCATATATGGGTTCtctacatatatattcct
	I miR-s	gaTAACTCTTCGTGTAGATGCGTtctctcttttgtattcc

AT1G72910	II miR-a	gaACGCATCTACACGAAGAGTTAtcaaagagaatcaatga
and	III miR*s	gaACACATCTACACGTAGAGTTTtcacaggtcgtgatatg
AT1G72940	IV miR*a	gaAAACTCTACGTGTAGATGTGTtctacatatatattcct

Uppercase letters = Coding sequences, lowercase letters = infusion/restriction enzyme sites.
